# Water Salinity Impacts Aggregation, Settling, and Deposition of Fluvial Sediment

**DOI:** 10.1021/acsenvironau.5c00134

**Published:** 2025-08-22

**Authors:** Philip J. Brahana, Bhuvnesh Bharti

**Affiliations:** Cain Department of Chemical Engineering, 5779 Louisiana State University, Baton Rouge, Louisiana 70803, United States

**Keywords:** river sediment, sediment transport, settling velocity, sedimentation kinetics, particle aggregation, turbidity, suspended particulate matter

## Abstract

Global wetlands have declined by 21–35% since the 18th century, losing approximately 1.3 million square miles. Infrastructure development, specifically, river channelization via levee construction, is a driver of this decline. In response, large-scale river diversion projects have been proposed to enhance sediment deposition and stabilize coastal wetlands. However, the role of aquatic chemistry in controlling the fluvial sediment deposition remains elusive. Here, we demonstrate that land formation by fluvial sediment deposition is intrinsically linked to wetland water salinity, which influences the sediment aggregation and settling kinetics. In laboratory experiments, Mississippi River sediments were exposed to a range of salinities that mimic the conditions in Louisiana wetlands. Our results show that higher ionic strength accelerates sediment aggregation and settling due to electrical double-layer compression while also reducing the packing density of deposited sediments, potentially impacting land stability. These findings point to the importance of incorporating salinity effects to optimize sediment diversion strategies.

## Graphical abstract

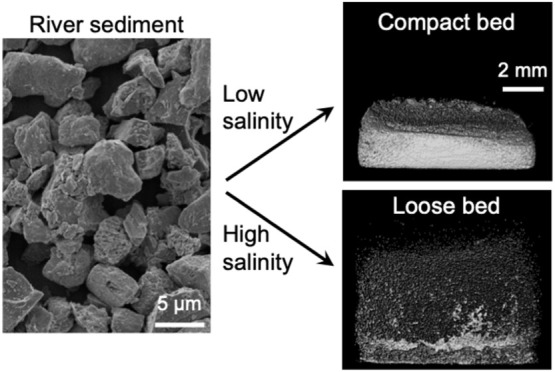

## 1 Introduction

Wetlands are dynamic ecosystems characterized by water-saturated soils, forming the interface of terrestrial and aquatic environments.^2^ They play critical roles in water purification,^3^ flood control,^4^ carbon sequestration,^5^ and supporting biodiversity.^6^ However, the alarming rate at which wetlands are disappearing worldwide highlights their extreme vulnerability.^7^ For example, the state of Louisiana in the United States alone loses ∼35 square miles of wetlands each year.^8–11^ The disappearance of wetlands is partly driven by natural geological cycles, including subsidence.^12^ However, human activities such as levee construction have reduced sediment delivery from the rivers to these “sediment-starved” wetlands.^13^ Wetland loss has adverse effects on the environment, the economy, and the communities in these regions. Environmentally, disappearing wetlands leads to the deterioration of coastal ecosystems, which often support endangered species such as the West Indian Manatee.^14^ Economically, wetland loss is expected to have substantial impacts on the seafood industry, which has an annual economic impact of over $2.4 billion for the State of Louisiana.^15–17^ For the communities that inhabit these regions, wetland loss increases vulnerability to extreme weather events such as hurricanes, storm surges, and flooding, as these wetlands once served as a natural buffer against such events.^18^ To address the growing challenge of wetland disappearance, efforts are underway to mitigate land loss through various restoration projects, including dredging,^19^ the implementation of living shorelines^20^ and proposed sediment diversions for gradual deposition of fluvial sediments.^21^

Sediment diversions are a targeted river management strategy that directs sediment-rich water into eroding wetlands and aids their restoration.^22,23^ These multibillion-dollar hydraulic engineering projects are aimed to counteract decades of land loss by reestablishing the hydrological balance in coastal wetlands. Initial reports indicate that the diversion projects can successfully deliver sediments and help in wetland restoration.^24,25^ However, these results come with some key caveats that govern the efficacy of the diversion projects. For example, it has been suggested that factors such as the elevation and placement of the diversion intake and the curvature of the river channel can influence the sediment capture efficiency.^26^ Another critical but often overlooked parameter in evaluating the efficacy of sediment diversions is the salinity of water in the wetlands. River diversions may introduce large volumes of freshwater into otherwise brackish wetlands.^27^ Such large freshwater inputs will not only alter the salinity of water in wetlands but also directly influence the settling and deposition of the sediments. In fact, a recent field study in Barataria Bay, Louisiana, demonstrated that salinity influences the settling velocity of estuarine sediments.^28^ Despite this, the mechanisms by which salinity impacts fluvial sediment aggregation and settling behaviors remain largely unexplored. Understanding the mechanisms by which the salinity of water influences the fluvial sediments is necessary to optimize the deposition and retention of sediment in coastal wetlands, reinforcing the idea that chemical and physical processes are interconnected across watersheds and should be considered in restoration efforts.^29,30^

Our laboratory study establishes a direct link between the aggregation, settling, and deposits of fluvial sediments and the surrounding aquatic chemistry. We collected sediment from the Mississippi River to represent the particulates deposited during Louisiana’s wetland restoration projects and created laboratory conditions that mimic the salinity levels found in these wetlands (Figure 1A). We quantify the impact of salinity on sediment aggregation, settling kinetics, and the characteristics of the sediment deposits.

**Figure 1:**
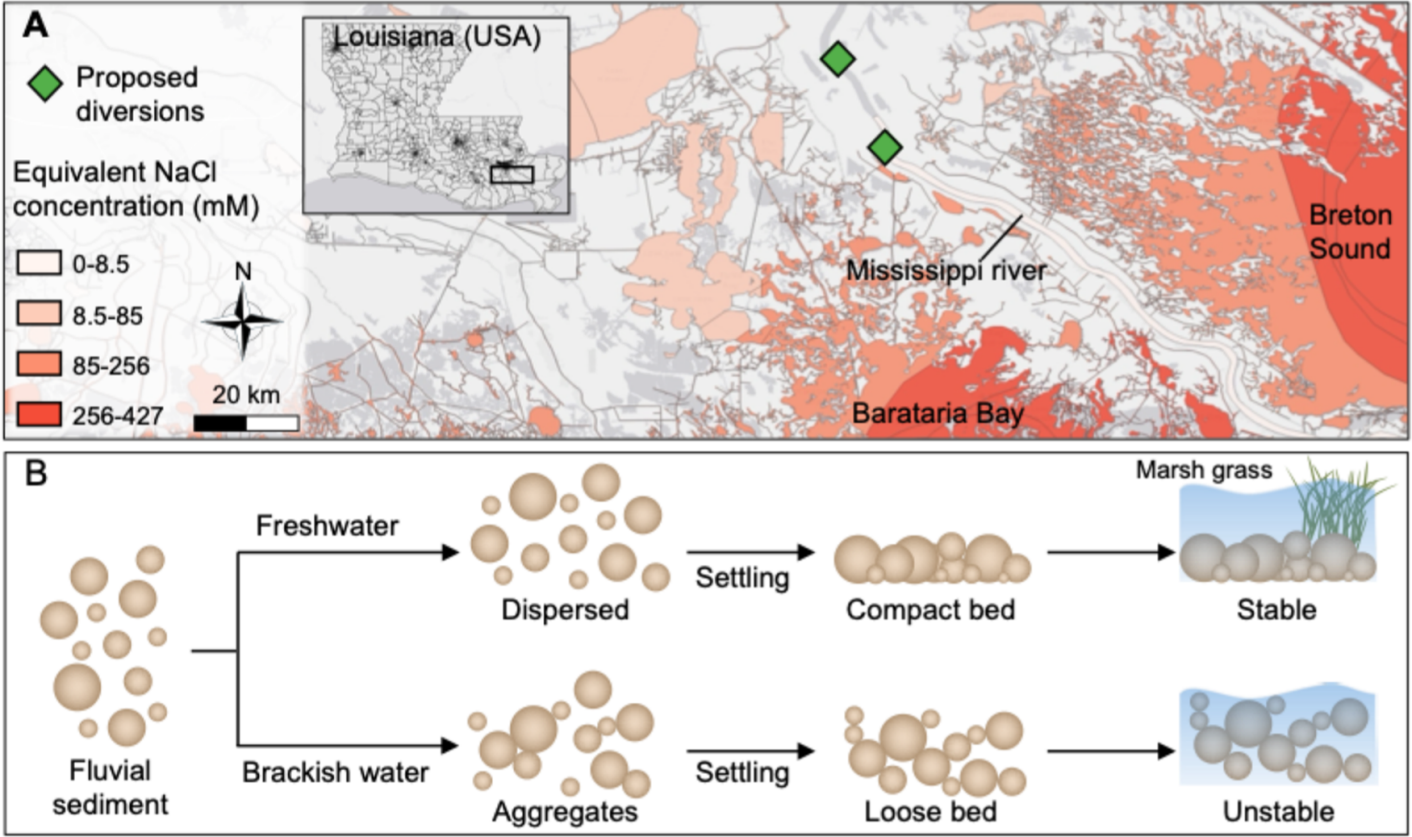
Water salinity variation in the Mississippi River Delta influences fluvial sediment. (A) A map of coastal Louisiana showing the existing salinity variations in the coastal estuaries during the winter of 2024. The green points represent the locations of the two proposed sediment diversions to feed the wetlands in the Barataria Bay and Breton Sound regions of the state. The salinity data was obtained from the National Oceanic and Atmospheric Administration (NOAA) Geoplatform^1^ and visualized in Quantum Geographic Information System software (QGIS 3.38.3). The equivalent NaCl concentrations (in mM) are estimated from the practical salinity units (PSU) based on ionic strength (see Section 3). (B) Schematic illustrating the contrasting aggregation behaviors of fluvial sediments under freshwater and brackish conditions. These differences in aggregate structure directly influence the sediment deposition, thereby affecting the land-building process.

## 2 Results and Discussion

### 2.1 Salinity-Driven Aggregation of Fluvial Sediment

Fluvial sediments encounter a range of salinity levels as they transition from river water into wetlands, potentially triggering significant changes in their aggregation behavior (Figure 1B). To investigate this, we collected sediment from the Mississippi River (30.361133, −91.235467) at a river stage of 22.7 ft (flood stage = 35 ft) using 5 L glass containers. Scanning electron microscope (SEM) image of the “as collected” sediment highlights their irregular shape and size in the range from tens of nanometers to tens of micrometers (Figure 2A). Prior to investigating the aggregation behavior, we removed the organic matter coating the sediment surfaces by digesting the samples in a 6% NaClO solution (pH 8) at room temperature for 24 h, followed by thorough rinsing with deionized water.^31^ Fourier transform infrared (FTIR) spectroscopy confirmed the removal of the organic layer, as indicated by the suppression of characteristic peaks at wavenumbers of 1632 and 3400 cm^–1^ (Figure S1). FTIR and X-ray diffraction measurements (Figures S1 and S3) further revealed that these sediments are rich in silicate-based minerals, primarily quartz and clays such as kaolinite and montmorillonite, which aligns with existing literature on Mississippi River sediments.^32,33^ We then investigated the aggregation behavior of these sediments in aqueous dispersions at pH ∼ 6 containing NaCl in concentration range 0–150 mM, which include the ionic strength of freshwater (<0.5 PSU ≡ <8 mM NaCl) and water found in the wetlands (5–15 PSU ≡ 80–250 mM NaCl) targeted for sediment diversions in southern Louisiana (Figure 1A and see Section 3). We acknowledge that introducing freshwater into saline wetlands will gradually reduce salinity; however, the initial stages of sediment deposition will be significantly influenced by the original salinity.

**Figure 2:**
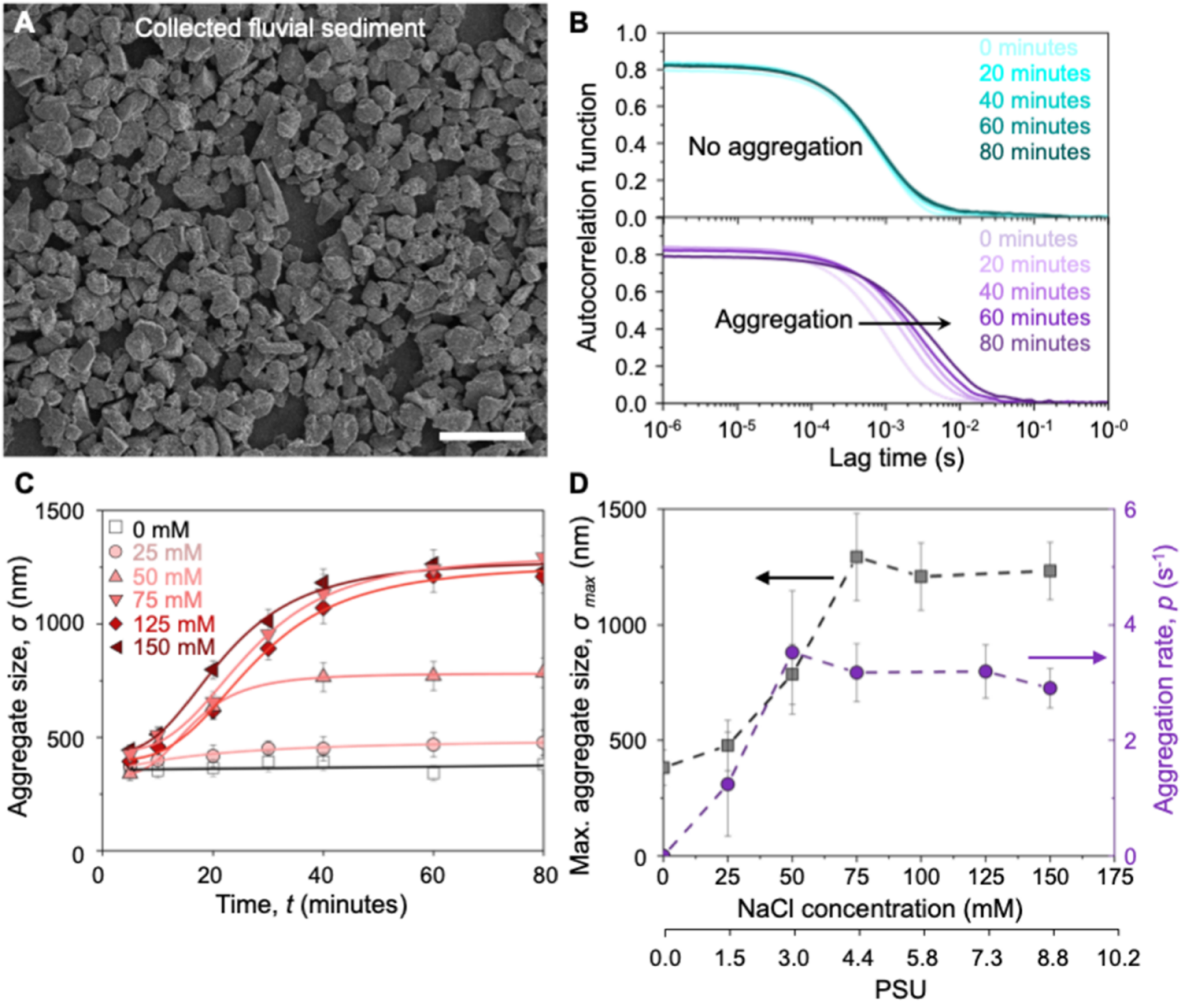
Role of salinity in directing the aggregation of fluvial sediment. (A) Scanning electron microscope (SEM) image of the collected fluvial sediment prior to washing and filtering. The scale bar is 25 μm. (B) The autocorrelation functions of filtered sediment dispersed in 0 mM NaCl (top panel) and 150 mM NaCl (bottom panel) obtained from dynamic light scattering (DLS). The shift in the autocorrelation function to longer lag times with increasing equilibration time for 150 mM NaCl indicates aggregation of sediment particles. (C) The measured size (effective diameter) of the sediment aggregates estimated from the autocorrelation functions shown in (B) as a function of equilibration time at NaCl concentrations between 0 and 150 mM. The solid lines in (C) are the fits to the experimental data using the logistic growth model. (D) The maximum aggregate size and aggregation rate as obtained by fitting the aggregate size data shown in (C) using the logistic growth model. The secondary *x*-axis shows the approximate PSU values that correspond to the NaCl concentrations on the primary *x*-axis. The dashed lines in (D) are the visual guides. The error bars in (C) correspond to the standard deviation of at least three measurements, and the error bars shown in (D) are correspondingly calculated using the propagation of error and uncertainty in the data fitting.

The structure of sediments and their aggregates critically influences their transport, deposition, and, consequently, the success of sediment diversion projects aimed at new land formation. The collected sediment exhibited a broad size distribution, ranging from the nanometer to micrometer scale (Figure 2A). To investigate the relationship between water salinity and sediment dispersion, we first filtered the washed sediment through a 0.45 μm syringe filter to isolate the smaller-sized fraction, followed by assessing their stability under varying NaCl concentrations (size ∼340 nm; Figure S2). We used dynamic light scattering (DLS) to monitor the temporal evolution of sediment size in suspensions containing 1.7 mg mL^–1^ of sediment with 0 and 150 mM (≡9 PSU) of added NaCl (25 °C, Figure 2A). DLS was performed on a Litesizer 500 (Anton Parr) equipped with a 658 nm laser at a backscattering angle of 175° to obtain time-correlation functions of the sediment dispersions at different salinities at pH ∼6. In the absence of added NaCl, the autocorrelation function (obtained from DLS) remained nearly unchanged over the experimental period of 80 min (Figure 2B), highlighting the stability of the sediments in water with no added NaCl. Whereas in water containing 150 mM NaCl, the autocorrelation function shifts to longer lag times, indicating slower dynamics, a signature of aggregation in the saline water.^34^ We determine the aggregate size (σ) in terms of its effective diameter by fitting the autocorrelation functions with a single exponential decay to extract the mean diffusivity, and then apply the Stokes–Einstein relation^35^ (Figure 2C). We find that the size of sediment aggregates remains nearly constant for 0 mM NaCl, and it doubles within 20 min for 150 mM NaCl (Figures 2B,C and S4), indicating aggregation under saline conditions. We further analyze the kinetics of the aggregate using a logistic growth model to extract the aggregation rate constant, *p* (Figure 2C). The logistic model provides a robust framework for describing the time evolution of aggregate size (σ), particularly in systems where growth exhibits an initial exponential phase followed by saturation due to limiting factors such as particle depletion. Mathematically, the logistic growth model represents aggregate size, σ, as a function of time *t* as^36,37^

1 $$\sigma =\sigma_{\min}+\sigma_{\max}/\left(1+\exp(-p(t-t_{0}\right))$$

where σ_min_ is the aggregate size formed after the initial 5 min taken for setting up the experiment, σ_max_ is the asymptotic or maximum aggregate size, which represents the upper limit of growth constrained by factors such as sediment availability, and *t*_0_ is the inflection point of the growth curve, indicating the time at which the aggregation rate is maximum. Analysis of aggregate size evolution over time at various salinities indicates that both σ_max_ and *p* increase as the concentration of NaCl rises from 0 to 150 mM in the sediment suspension (Figure 2D). Experiments with unwashed fluvial sediments that retain their natural organic coatings (Figure S5) confirm that salinity-dependent aggregation persists, indicating that high salinity promotes aggregation even in the presence of organic matter. This observation is further supported by ζ potential measurements taken before and after the washing process, which show only a minor decrease in the ζ potential following organic matter removal (Figure S6). These findings highlight the critical role of ionic strength in controlling the aggregating behavior of sediment in aqueous environments.

Sediment aggregation results from the interplay between electrical double-layer repulsion and van der Waals attraction, as defined by Derjaguin–Landau–Verwey–Overbeek (DLVO) theory.^38^ In our study at pH 6, the sediments exhibit a ζ potential of −20 mV, attributable to the presence of chemical functional groups such as carboxyl and silanol groups on the sediment surface.^39,40^ This charge creates an electrical double layer around the particles.^41^ At a low ionic strength, the extended diffuse layer produces strong osmotic repulsion that prevents aggregation. As the ionic strength increases, enhanced charge screening compresses the electrical double layer by reducing the Debye length (and decreasing the ζ potential), which weakens the repulsive forces and allows van der Waals attractions to dominate. This shift makes the sediments “stickier”, leading to the formation of loose aggregates through a diffusion-limited “hit-and-stick” mechanism.^42^ For the finer particles in the filtered fraction analyzed via DLS, Brownian motion plays an important role in enabling such diffusion-limited aggregation. However, for the coarser sediment particles used in the settling experiments (discussed later), the diffusion effects are likely minimized due to their larger size. In this larger size regime, external forces such as fluid shear would primarily drive aggregation, resulting in fractal aggregates with large volumes that settle to form beds of a low packing fraction (see following section). Note that given the large size distribution of the collected sediment, both Brownian diffusion and fluid shear would drive the aggregation kinetics and thus influence the morphology of the settled sediment bed. In low ionic strength environments, a repulsive barrier would allow for local particle rearrangements in the settling sediment, leading to a higher sediment packing fraction in the bed.

### 2.2 Impact of Salinity on Settling of Fluvial Sediment

The aggregated state of the fluvial sediment plays a critical role in the settling and packing of the sediment bed. As demonstrated, the sediment existed in a dispersed form at low salinity (freshwater) and as aggregates under the salinity of brackish water (Figure 2). To quantify the influence of the dispersed state of the sediment on its settling, we monitor the change in turbidity of unfiltered sediment dispersions in varying salinity conditions over a period of ∼50 min (Figure 3A). The change in turbidity over time is an indirect method for observing sediment settling as the measured turbidity scales linearly with the log­(transmittance), hence concentration of suspended particles in the media (see Section 3).^43^ Upon settling, the concentration of the sediments in the dispersion (and hence turbidity) decreases with time, which enables the determination of the effect of changing the salinity on the kinetics of settling. In a typical experiment, fluvial sediment was suspended at 3 mg mL^–1^ in solutions with NaCl concentrations ranging from 0 to 150 mM. After allowing the aggregation process to proceed for 24 h, the suspension was gently transferred to a fresh cuvette for settling analysis. Turbidity was measured at a height of 4 cm in a rectangular quartz cuvette with a 1 cm path length using Anton Paar’s Litesizer 500.

**Figure 3:**
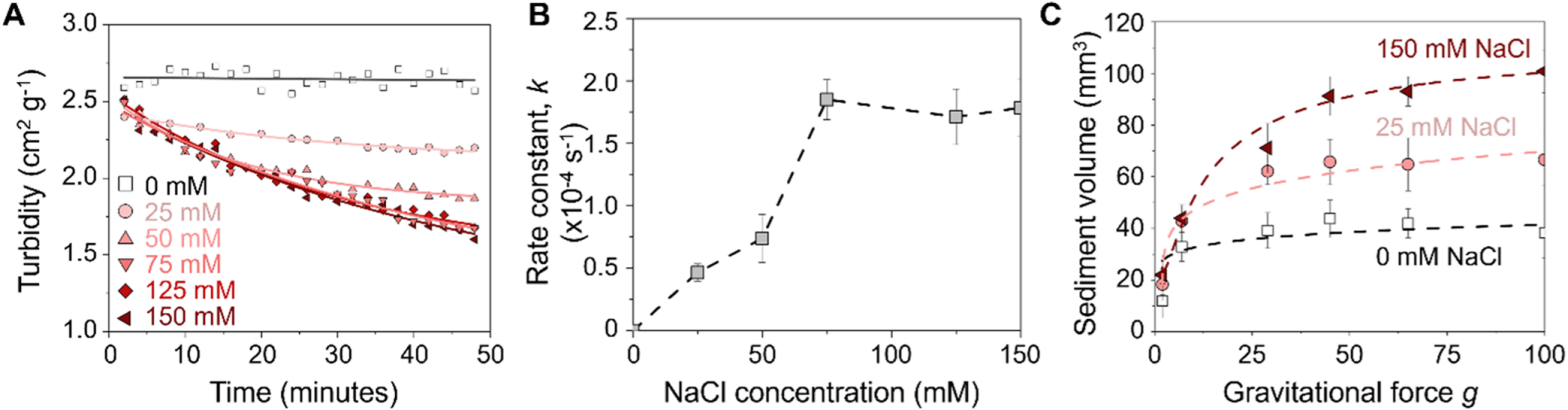
Impacts of salinity on the settling of fluvial sediment. (A) The gradual decrease in turbidity for sediment suspension with time, containing NaCl concentrations between 0 and 150 mM. The turbidity values were calculated from the optical transmittance (see Section 3). The points are the measured data, and lines represent the fit to the experimental data using the pseudo-first-order settling kinetics. (B) Change in the sediment settling rate constant extracted from the pseudo-first-order kinetics shown in (A). (C) The settled sediment volume as a function of applied centrifugal acceleration, *g*. The dashed lines in (C) and (D) are added for visual guidance. In (B) and (C), the error bars correspond to the standard deviation of at least three measurements.

The rate of decrease in turbidity increases with increasing dispersion salinity, indicating a rapid settling of sediment under saline conditions. The turbidity of suspension of sediments in freshwater remains nearly constant for up to 6 h, suggesting prolonged dispersion of sediment (Figure 3A). In contrast, we observe a sharp decline in turbidity within the first hour in brackish water conditions, indicating rapid sediment settling. The turbidity under saline conditions stabilizes at a nonzero value, suggesting that a portion of the sediment remains suspended, presumably due to the presence of small sediment particles. We quantify the change in turbidity by modeling the settling process using a pseudo-first-order rate equation,^44,45^ where turbidity is approximated to be influenced solely by the concentration of suspended sediment, i.e., turbidity is directly proportional to the remaining concentration of sediments in the suspension (see Section 3 and Figure S7). Using this approximation, we find that the sediment settling rate increases from 0 to 1.7 × 10^–4^ s^–1^ upon increasing the concentration of NaCl from 0 to 75 mM and thereby remains nearly constant with further increase in salinity (Figure 3B). To further evaluate the suitability of the kinetic model, we compared pseudo-first-order fits with zero- and second-order kinetic models, as summarized in Table S1. All models yield comparable trends and goodness-of-fit metrics, highlighting the complexity of the intertwined aggregation and settling processes. Based on the salinity, sediments may strongly interact and aggregate during settling, causing the settling velocity to vary with time and making such analysis nontrivial. Here we use the pseudo-first-order model as a simplified yet effective empirical approach to characterize sediment settling behavior under varying salinities. Further work is required to identify a precise kinetic model that accurately captures the settling behavior of aggregating sediments at different settling time regimes. The change in the settling kinetics of the sediment with increasing salinity is attributed to the formation of aggregates, which align with the existing literature on the aggregation of charge-stabilized colloidal particles upon increasing ionic strength.^46–48^ We further estimate the settling velocity of the sediment aggregates using the Stokes relation with sediment mass density^49^ of ∼2.5 g cm^–3^, and effective size shown in Figure 2. The sedimentation velocity shows an increase from 0.1 to 1.2 mm h^–1^ upon increasing the concentration of NaCl from 0 to 150 mM.

The volume of the settled sediment bed is linked to the aggregated state of the sediment particles. We quantified the effect of centrifugal force on the volume of settled sediment particles as a function of dispersion salinity in the range 0 to 150 mM NaCl (Figure 3C). We measured the volume of a sediment bed formed from 5 mL of a 10 mg mL^–1^ suspension under varying centrifugal accelerations (and thus force) for 10 min. In this study, centrifugal acceleration is expressed in multiples of gravitational acceleration (*g*) rather than as a force. The volume of the settled sediments increases with applied centrifugal force across all salinities. It initially rises steeply before approaching an asymptotic limit. This initial increase indicates a high rate of sediment settling from the suspension, and the plateau at higher *g* values suggests that further compaction is minimal once all sediment attains the jammed state. For example, for 100*g*, the value of sediment volume at a 150 mM NaCl solution is nearly three times that at 0 mM NaCl. Assuming all sediment particles settle after 100*g* centrifugal acceleration for 10 min, we can estimate the packing fraction (ϕ) of the sediment particles using their mass density. We find that the packing fraction of sediment at 100*g* decreases from 0.54 to 0.20 upon increasing the concentration of NaCl from 0 to 150 mM. This difference supports our hypothesis that elevated salinity promotes the formation of fractal-like, loosely packed aggregates. Albeit the fractal structure of the aggregates would limit the full densification, the aggregates are partially compressed under centrifugal force. Hence, the values of ϕ obtained from centrifugation experiments are not directly comparable to those from natural settling processes at 1*g*. We further characterize the 3D sediment structure in the packed bed using microcomputed tomography (micro-CT).

### 2.3 Structure and Pattern of Sediment Deposits

The sediment settled to form a dense layer in the absence of added NaCl but formed voluminous deposits at 150 mM NaCl. To characterize the internal structure of these deposits, we performed micro-CT using X-rays. It is a nondestructive imaging technique that provides high-resolution 3D representations of materials, enabling detailed analysis of internal structures, density variations, and sediment distributions (Figure 4A). We performed micro-CT on sediments deposited from 3 mL of 80 mg mL^–1^ suspensions containing 0 and 150 mM NaCl within a polystyrene rectangular cuvette. The sediment particles were allowed to aggregate and settle at 1*g* for 48 h prior to capturing a series of X-ray projections over a volume of 5 cm^3^ with a spatial resolution of 5.9 μm (Figure 4B,C). The 3D reconstruction of these projections reveals that the sediment packing is strongly influenced by salinity. Under freshwater conditions, the sediment forms a compact layer, whereas under saline conditions, it develops a more porous and loosely packed structure. The packing fraction (ϕ) decreases from 0.52 to 0.02 as NaCl concentration increases from 0 to 150 mM, corroborating our sediment compression study (Figure 3C). It is important to note that the ϕ values from centrifugation (measured at 100*g*) and micro-CT (measured at 1*g*) are not quantitatively comparable. At 0 mM NaCl, the packing fraction estimates are similar from both methods, as sediments settle and pack densely. However, at 150 mM NaCl, ϕ values from centrifugation are significantly higher due to forced compaction, while micro-CT reflects the natural, loosely packed structure of the aggregates. The low packing fraction (∼0.02) at 1*g* and high salinity is due to the settling of the fractal-like aggregates that resist compaction. At 0 mM NaCl, similar packing fractions under 1*g* and 100*g* arise from different settling mechanisms but yield the same closed-packed structure when sediment aggregation is absent. The probability distribution of aggregate volume within the sediment (from micro-CT) also shows a shift to higher values upon increasing the concentration of NaCl from 0 to 150 mM (Figure 4D). Such a shift demonstrates that saline conditions promote the formation of large, low-density aggregates, consistent with our aggregate size measurements shown in Figure 2.

**Figure 4:**
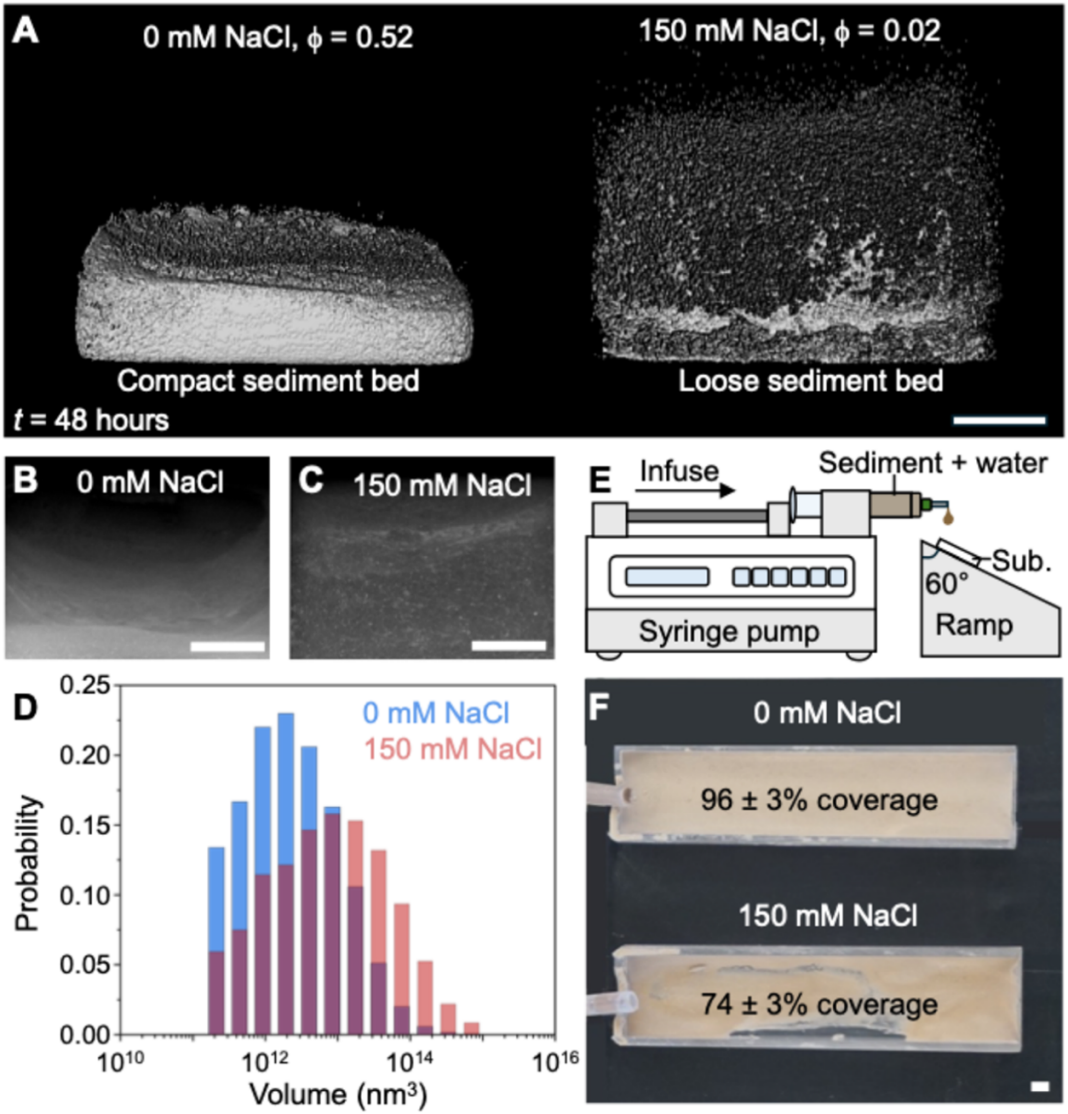
Influence of salinity on the sediment deposits. (A) 3D reconstruction of the deposited sediment layer obtained from micro-CT. Snapshots of the raw images from which the 3D reconstruction was generated, showing the settled sediments from dispersions containing (B) 0 mM NaCl and (C) 150 mM NaCl. (D) The probability distribution of the aggregate volume as determined by the micro-CT imaging. The average volume of the aggregate increased upon increasing the salinity. (E) Schematic representation of the experimental setup for the continuous flow experiments. (F) The dried layer of sediment deposited on an inclined substrate after 2 h of continuous flow of sediment suspension containing 0 and 150 mM of added NaCl. The reported surface coverage for the 150 mM NaCl condition is 74 ± 3%, indicating that the actual coverage likely falls within the range of 71–77%, reflecting the measurement uncertainty. The scale bars in (A)–(C) and (F) are 2 mm.

The salinity of water influences the structure of the sediment aggregates and deposition patterns from flowing water. We qualitatively assess how sediment aggregate structure influences the deposition and stability of settled sediment by exposing a continuous flow of sediment-rich water onto a roughened plastic substrate on an inclined plane (angle of inclination 60°; Figure 4E,F). We dispersed 50 mg mL^–1^ of washed fluvial sediment in solutions containing 0 and 150 mM NaCl. The suspensions were delivered via syringes connected to a pump operating at 0.1 mL min^–1^ for 2 h. After the equilibration period, each substrate is dried and imaged for further analysis. We find nearly 99% of the substrate was covered by sediment from the 0 mM NaCl, while only about 74% was covered under saline conditions of 150 mM NaCl (Figure 4E,F). These qualitative findings of flow deposition experiments combined with micro-CT imaging show that water salinity plays a critical role in determining the internal structure of sediment deposits and highlight the need to consider environmental salinity in sediment diversion models.

Our results show that high salinity conditions promote the formation of loose, fractal aggregates that settle more rapidly but form beds with lower packing fractions. In contrast, low salinity conditions result in slower settling but more compact bed structures. From a restoration standpoint, this suggests a trade-off between rapid sediment deposition and long-term soil consolidation or stability. However, translating these findings to natural wetland environments requires the consideration of additional complexities. Real-world wetlands exhibit spatial gradients in salinity, sediment supply, flow conditions, and biological activity, all of which can influence sediment behavior and deposition patterns over varying temporal and spatial scales. Furthermore, the time scales of sediment transport and wetland evolution in the field also span much longer durations than those accessible in laboratory experiments. Therefore, while our results establish a mechanistic understanding relevant to sediment dynamics in brackish and freshwater conditions, field-based studies incorporating ecosystem complexity and environmental variability are essential to validate and extend these findings. Such integrative approaches will be critical for applying fundamental sedimentation processes toward effective wetland restoration and management strategies.

In summary, our study establishes a direct link between the aggregation, settling, and deposition of fluvial sediments and the surrounding aquatic chemistry, specifically, water salinity. We show that ionic strength strongly influences sediment aggregation kinetics, with higher salinity accelerating aggregation and promoting the formation of larger-sized aggregates. As a result, the settling behavior of these aggregates varies; brackish and saline conditions lead to rapid settling, while freshwater suspensions remain dispersed for extended periods. Although deposition occurs under all salinity conditions, high saline environments yield more voluminous, patchy deposits with lower packing fractions, whereas freshwater conditions produce compact, uniformly packed layers. These results highlight the critical role of aquatic chemistry in controlling sediment transport and deposition, emphasizing that salinity must be considered when planning large-scale sediment diversions aimed at land-building. Further lab and field-based studies are needed to validate these findings under natural conditions, including in the presence of biofilms,^50^ microplastics^51,52^ and other anthropogenic pollutants,^53^ which may also influence fluvial sediment behavior. Such insights will be crucial for optimizing sediment diversion strategies to enhance coastal restoration efforts across the world, such as in the state of Louisiana.

## 3 Methods

### 3.1 Sediment Characterization

The fluvial sediment and sediment aggregates were characterized by dynamic light scattering (DLS), Fourier transform infrared spectroscopy (FTIR), X-ray diffraction (XRD), and ζ potential measurements. DLS was performed on a Litesizer 500 (Anton Parr) equipped with a 658 nm laser to obtain time-correlation functions of the sediment dispersions at different salinities at a backscattering angle of 175°. The FTIR spectra of the sediment was obtained with a Bruker Alpha FTIR instrument using a monolithic diamond crystal ATR accessory. The instrument was blanked with air, and measurements were scanned 32 times per spectrum at a 4 cm^–1^ resolution. The XRD was carried out on a Bruker D8 X-ray diffractometer. The scans were repeated three times on three different samples, yielding consistent results. The ζ potential measurements were done in the Univette cuvette (Anton Parr), applying a 10 V current across the suspension and monitoring its electrophoretic mobility using the laser with a wavelength of 658 nm.

### 3.2 Turbidity Measurements

Turbidity was determined from the optical transmittance (Tr), measured in a 1 cm quartz cuvette with a Litesizer 500 instrument (Anton Parr). Optical transmittance values were converted to turbidity *T*_*t*_ at time *t* using the following expression: *T*_*t*_ = −log­(Tr)/*lC*_0_, where *C*_0_ is the initial total concentration of fluvial sediment in the suspension and *l* is the optical path length.^38^

### 3.3 Pseudo-First-Order Model for Settling Rate

To evaluate the settling behavior of particles under pseudo-first-order kinetics, the concentration–time data was linearized using the integrated form of the pseudo-first-order rate law, which assumes that the rate of change in concentration is proportional to the remaining concentration (*C*_*t*_) at time *t*. Assuming that turbidity (*T*_*t*_) ∝ *C*_*t*_, the rate equation can be written as ln­(*T*_0_/*T*_*t*_) = *kt*, where *T*_0_ is the initial turbidity at time *t* = 0, *T*_*t*_ is the turbidity at time *t*, and *k* is the sediment settling rate constant. The plot of ln­(*T*_0_/*T*_*t*_) vs *t* was constructed, and the rate constant *k* was extracted from the slope of the resulting linear fit (Figure S7). A linear relationship with a high correlation coefficient (*R* ^2^ > 0.95) was used as an indicator of pseudo-first-order kinetics. The slope of the ln­(*T*_0_/*T*_*t*_) vs *t* plot corresponds to the sediment settling rate constant with units of inverse time (s^–1^).

### 3.4 Micro-CT Using X-rays

The micro-CT was performed using HeliScan Micro-CT (ThermoFisher Scientific, USA). The settled sediment samples are helically scanned at a voxel resolution of 5.9 μm using 4420 projections in 2 h. The scanning parameters are a 65 mA current, 90 kV voltage, and 0.8 s exposure time. The output from the micro-CT is analyzed in Avizo 2024.2.

### 3.5 Scanning Electron Microscopy (SEM)

Scanning electron microscopy was done with a Quanta 3D DualBeam FEG FIB-SEM with an accelerating voltage of 5 kV. The settled sediment samples from the continuous flow experiments were allowed to dry overnight before being coated with a 5 nm layer of platinum to prevent charging.

### 3.6 PSU to Equivalent NaCl Concentration Conversion

To estimate the equivalent NaCl concentration in millimolar (mM) from the National Oceanic and Atmospheric Geoplatform data set, which is given in practical salinity units (PSU), a mass-based approximate conversion was applied. Given that 1 PSU is approximately equivalent to 1000 ppm, the salinity value in PSU was first converted to ppm. Assuming NaCl as the primary contributor to salinity, the NaCl concentration in millimolar (mM) was estimated by dividing the ppm value by the molar mass of NaCl (58.44 g mol^–1^). This approach provides an equivalent NaCl concentration while recognizing that natural water samples contain additional dissolved salts. Note that the ionic strength and salinity represented by the equivalent NaCl concentration underestimate the actual values of ionic strength in the field, which would also include multivalent ions.

## Supplementary Material



## Notes

FTIR analysis of unwashed and washed sediment; DLS particle size and aggregation at varying salinities; XRD mineral composition; ζ potential measurements; turbidity decay kinetics; and comparison of zero-, pseudo-first-, and second-order kinetic model fits (PDF)

## References

[1] NOAA Office for Coastal Management Planned Data Acquisition for Aerial Imagery, 2024. https://www.fisheries.noaa.gov/inport/item/48838 (accessed Feb 19, 2025).

[2] Cronk, J. K. ; Fennessy, M. S. Wetland Plants: Biology and Ecology; CRC Press, 2016.

[3] Wu H., Wang R., Yan P., Wu S., Chen Z., Zhao Y., Cheng C., Hu Z., Zhuang L., Guo Z. (2023). Constructed wetlands
for pollution control. Nat. Rev. Earth Environ..

[4] Zhu Z., Vuik V., Visser P. J., Soens T., van Wesenbeeck B., van de Koppel J., Jonkman S. N., Temmerman S., Bouma T. J. (2020). Historic storms
and the hidden value of coastal wetlands
for nature-based flood defence. Nat. Sustainability.

[5] Were D., Kansiime F., Fetahi T., Cooper A., Jjuuko C. (2019). Carbon sequestration
by wetlands: a critical review of enhancement measures for climate
change mitigation. Earth Syst. Environ..

[6] Denny P. (1994). Biodiversity
and wetlands. Wetlands Ecol. Manage..

[7] Osland M. J., Chivoiu B., Enwright N. M., Thorne K. M., Guntenspergen G. R., Grace J. B., Dale L. L., Brooks W., Herold N., Day J. W., Sklar F. H., Swarzenzki C. M. (2022). Migration
and transformation of coastal wetlands in response to rising seas. Sci. Adv..

[8] Bourne J. (2000). Louisiana’s
vanishing wetlands: Going, going. Science.

[9] Tibbetts, J. Louisiana’s Wetlands: A Lesson in Nature Appreciation; National Institute of Environmental Health Sciences, 2006. 10.1289/ehp.114-a40 PMC1332684 16393646

[10] Stokstad E. (2005). Louisiana’s
Wetlands Struggle for Survival. Science.

[11] Zhong B., Xu Y. J. (2011). Risk of Inundation to Coastal Wetlands and Soil Organic Carbon and
Organic Nitrogen Accounting in Louisiana, USA. Environ. Sci. Technol..

[12] Morton, R. A. ; Bernier, J. C. ; Barras, J. A. ; Ferina, N. F. Rapid Subsidence and Historical Wetland Loss in the Mississippi Delta Plain: Likely Causes and Future Implications; U. S. Geological Survey, 2005.

[13] Walker H. J., Coleman J. M., Roberts H. H., Tye R. S. (1987). Wetland loss in
Louisiana. Geografiska Annaler: Ser. A, Phys.
Geogr..

[14] Fertl D., Schiro A., Regan G., Beck C. A., Adimey N., Price-May L., Amos A., Worthy G. A., Crossland R. (2005). Manatee occurrence
in the northern Gulf of Mexico, west of Florida. Gulf Caribb. Res..

[15] Bigford T. E. (1991). Sea-level
rise, nearshore fisheries, and the fishing industry. Coastal Manage..

[16] Wall G. (1998). Implications
of global climate change for tourism and recreation in wetland areas. Clim. Change.

[17] Smith, M. ; Steward, G. T. ; Massaua, M. ; Atwood, D. ; Lesnick, M. ; Hymel, T. An Economic Development Strategy for Louisiana’s Coastal Seafood Industry; NOAA, 2020. https://repository.library.noaa.gov/view/noaa/36887 (accessed Aug 15, 2025).

[18] Sun F., Carson R. T. (2020). Coastal wetlands
reduce property damage during tropical
cyclones. Proc. Natl. Acad. Sci. U.S.A..

[19] Suedel B. C., McQueen A. D., Wilkens J. L., Saltus C. L., Bourne S. G., Gailani J. Z., King J. K., Corbino J. M. (2021). Beneficial use of
dredged sediment as a sustainable practice for restoring coastal marsh
habitat. Integr. Environ. Assess. Manage..

[20] Craton A. (2022). Calling All
Oysters: An Analysis of Living Shorelines, Legal Impediments, and
Louisiana’s Land Loss Crisis. LSU J.
Energy Law Resour..

[21] Allison M. A., Meselhe E. A. (2010). The use of large
water and sediment diversions in the
lower Mississippi River (Louisiana) for coastal restoration. J. Hydrol..

[22] Xu K., Bentley S. J., Day J. W., Freeman A. M. (2019). A review of sediment
diversion in the Mississippi River Deltaic Plain. Estuarine, Coastal Shelf Sci..

[23] Coastal Protection and Restoration Authority of Louisiana . Louisiana’s Comprehensive Master Plan for a Sustainable Coast; Coastal Protection and Restoration Authority of Louisiana, 2017.

[24] White J. R., Couvillion B., Day J. W. (2023). Coastal wetland area change for two
freshwater diversions in the Mississippi River Delta. Ecol. Eng..

[25] White J. R., Fulweiler R. W., Li C. Y., Bargu S., Walker N. D., Twilley R. R., Green S. E. (2009). Mississippi River
Flood of 2008:
Observations of a Large Freshwater Diversion on Physical, Chemical,
and Biological Characteristics of a Shallow Estuarine Lake. Environ. Sci. Technol..

[26] Meselhe E. A., Sadid K. M., Allison M. A. (2016). Riverside
morphological response
to pulsed sediment diversions. Geomorphology.

[27] Henkel T. K., Freeman A. M., Lindquist D. C., Pahl J. W., Troutman J. P. (2023). Lessons
learned from 30-years of operation of the caernarvon freshwater diversion,
Louisiana USA. Ocean Coastal Manage..

[28] McDonell M., Strom K., Nittrouer J., Mariotti G. (2024). Quantifying mud settling
velocity as a function of turbulence and salinity in a deltaic estuary. Cont. Shelf Res..

[29] Wohl E., Angermeier P. L., Bledsoe B., Kondolf G. M., MacDonnell L., Merritt D. M., Palmer M. A., Poff N. L., Tarboton D. (2005). River restoration. Water Resour.
Res..

[30] Wohl E. (2025). Conceptualizing
River Floodplains. Earth’s Future.

[31] Mikutta R., Kleber M., Kaiser K., Jahn R. (2005). Organic matter removal
from soils using hydrogen peroxide, sodium hypochlorite, and disodium
peroxodisulfate. Soil Sci. Soc. Am. J..

[32] Piper D. Z., Ludington S., Duval J. S., Taylor H. E. (2006). Geochemistry of
bed and suspended sediment in the Mississippi river system: Provenance
versus weathering and winnowing. Sci. Total
Environ..

[33] Taggart M. S., Kaiser A. (1960). Clay mineralogy
of Mississippi River deltaic sediments. Geol.
Soc. Am. Bull..

[34] Bharti B., Fameau A.-L., Rubinstein M., Velev O. D. (2015). Nanocapillarity-mediated
magnetic assembly of nanoparticles into ultraflexible filaments and
reconfigurable networks. Nat. Mater..

[35] Pete A. J., Lee J. G., Benton M. G., Bharti B. (2023). Chitosan-Coated Lignin
Nanoparticles Enhance Adsorption and Proliferation of Alcanivorax
borkumensis at the Hexadecane–Water Interface. ACS ES&T Eng..

[36] Chassagne C., Safar Z. (2020). Modelling flocculation:
Towards an integration in large-scale sediment
transport models. Mar. Geol..

[37] Safar Z., Deng Z., Chassagne C. (2023). Applying a
logistic growth equation
to model flocculation of sediment in the presence of living and dead
organic matter. Front. Mar. Sci..

[38] Bharti B., Meissner J., Findenegg G. H. (2011). Aggregation
of silica nanoparticles
directed by adsorption of lysozyme. Langmuir.

[39] Jada A., Akbour R. A., Douch J. (2006). Surface charge
and adsorption from
water onto quartz sand of humic acid. Chemosphere.

[40] Kubicki J., Schroeter L., Itoh M., Nguyen B., Apitz S. (1999). Attenuated
total reflectance Fourier-transform infrared spectroscopy of carboxylic
acids adsorbed onto mineral surfaces. Geochim.
Cosmochim. Acta.

[41] Israelachvili, J. N. Intermolecular and Surface Forces; Academic Press, 2011.

[42] Witten T.
A., Sander L. M. (1983). Diffusion-limited
aggregation. Phys. Rev. B.

[43] Matos T., Martins M., Henriques R., Goncalves L. (2024). A review of
methods and instruments to monitor turbidity and suspended sediment
concentration. J. Water Process Eng..

[44] Precious
Sibiya N., Rathilal S., Kweinor Tetteh E. (2021). Coagulation
Treatment of Wastewater: Kinetics and Natural Coagulant Evaluation. Molecules.

[45] Patel R., Brahana P. J., Bharti B. (2024). Enhanced Removal of
Anionic Pollutants
Using Active Propulsion of Patchy ZIF-8 Microparticles in Electric
Field. ACS Appl. Eng. Mater..

[46] Bouyer F., Robben A., Yu W. L., Borkovec M. (2001). Aggregation of colloidal
particles in the presence of oppositely charged polyelectrolytes:
effect of surface charge heterogeneities. Langmuir.

[47] French R.
A., Jacobson A. R., Kim B., Isley S. L., Penn R. L., Baveye P. C. (2009). Influence of ionic
strength, pH, and cation valence
on aggregation kinetics of titanium dioxide nanoparticles. Environ. Sci. Technol..

[48] Bharti B., Meissner J., Klapp S. H., Findenegg G. H. (2014). Bridging
interactions of proteins with silica nanoparticles: The influence
of pH, ionic strength and protein concentration. Soft Matter.

[49] Czuba J. A., Straub T. D., Curran C. A., Landers M. N., Domanski M. M. (2015). Comparison
of fluvial suspended-sediment concentrations and particle-size distributions
measured with in-stream laser diffraction and in physical samples. Water Resour. Res..

[50] Pete A. J., Brahana P. J., Bello M., Benton M. G., Bharti B. (2023). Biofilm Formation
Influences the Wettability and Settling of Microplastics. Environ. Sci. Technol. Lett..

[51] Al
Harraq A., Brahana P. J., Arcemont O., Zhang D., Valsaraj K. T., Bharti B. (2022). Effects of Weathering on Microplastic
Dispersibility and Pollutant Uptake Capacity. ACS Environ. Au.

[52] Brahana P., Zhang M., Nakouzi E., Bharti B. (2024). Weathering
influences
the ice nucleation activity of microplastics. Nat. Commun..

[53] Brahana P. J., Al Harraq A., Saab L. E., Roberg R., Valsaraj K. T., Bharti B. (2023). Uptake and
release of perfluoroalkyl carboxylic acids
(PFCAs) from macro and microplastics. Environ.
Sci.: Processes Impacts.

