# Supplementary material for: Water Salinity Impacts Aggregation, Settling, and Deposition of Fluvial Sediment
Source: ACS Environ Au. 2025 Aug 22;5(6):616–24. doi: 10.1021/acsenvironau.5c00134 (PMC12635937; doi:10.1021/acsenvironau.5c00134)
Supplement: Supplementary file 1 [file vg5c00134_si_001.pdf]

Supplementary Information for

Water salinity impacts aggregation, settling, and deposition of fluvial sediment

Philip J. Brahana and Bhuvnesh Bharti*

Cain Department of Chemical Engineering, Louisiana State University, Baton Rouge, LA 70803, USA

*corresponding author's email address: bbharti@lsu.edu

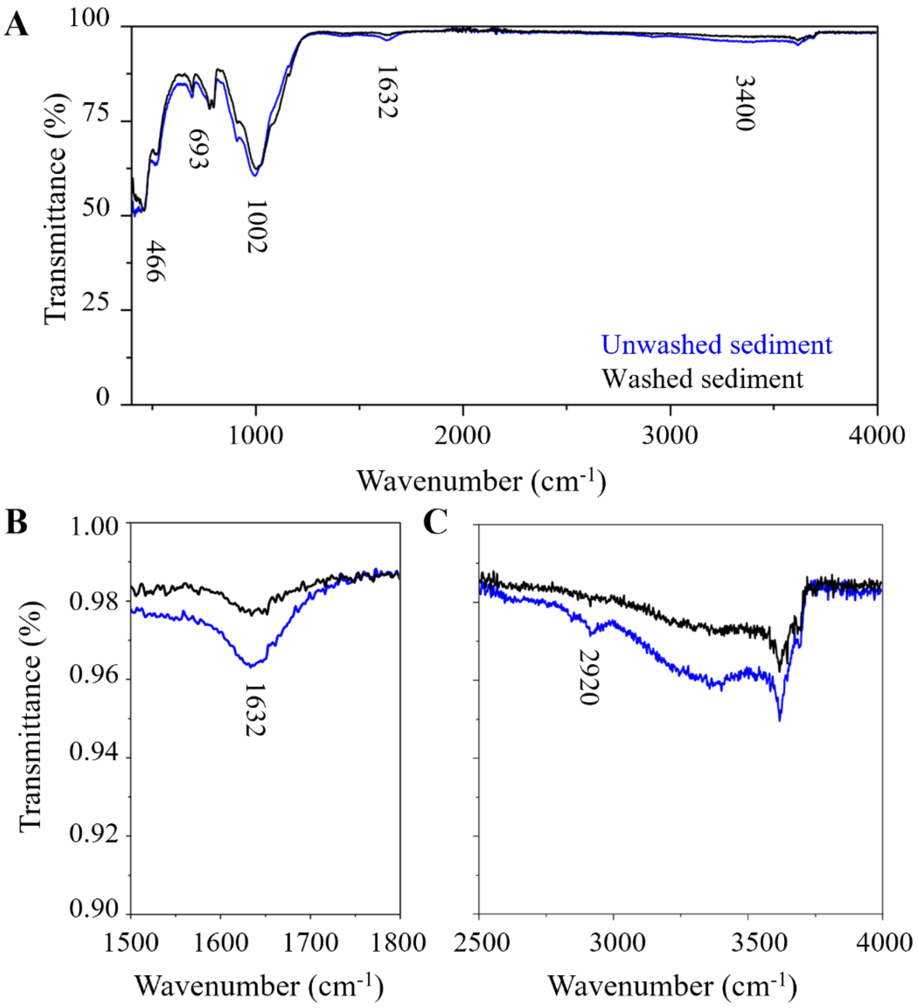

Figure S1: Fourier transform infrared spectroscopy (FTIR) of the unwashed (blue) and washed/treated (black) fluvial sediment. (a) Characteristic peaks are labeled at 466, 693, 1002, 1632 and 3400-3700 cm^{-1}. Vibrational peaks from 3400-3700, 1632 and 1002 cm^{-1} are associated with oxygen containing functional groups such as O-H, C=O and C-O, respectively[1]. Additionally, the peak at 693 cm^{2} is indicative of C-O-C bending. The intensity of the bands at 466 and 1002 is also associated with Si-O stretching vibration and bending vibration[1]. (b) The reduction of the and carbonyl peak (1500-1800 cm^{-1}). (c) The reduction of the -OH shoulder (3000-3700 cm^{-1}) and the aliphatic C-H peak (2920 cm^{-1}) between the unwashed and washed sediments highlights the effectiveness of the washing in removing the surface organic surface from the sediments. The y-axis in (b)and (c) are the identical.

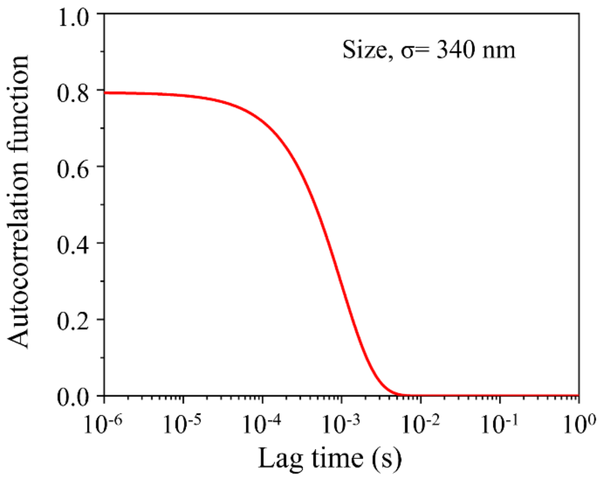

Figure S2: Autocorrelation function of the washed and filtered fluvial sediment. The data was fit to a single-decay exponential function to extract the sediment diameter of 340 nm.

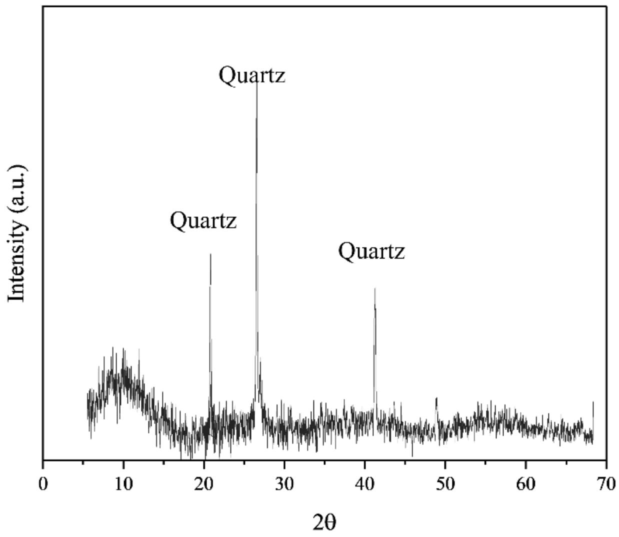

Figure S3: X-ray diffraction (XRD) pattern of fluvial sediment. The XRD pattern shows characteristic peaks for quartz, indicating a high silica content composition[2-5]. The diffraction intensity (a.u.) is plotted against the 2θ angle, with prominent peaks corresponding to the crystalline structure of quartz. The presence of these peaks suggests that silica-rich minerals dominate the sediment composition.

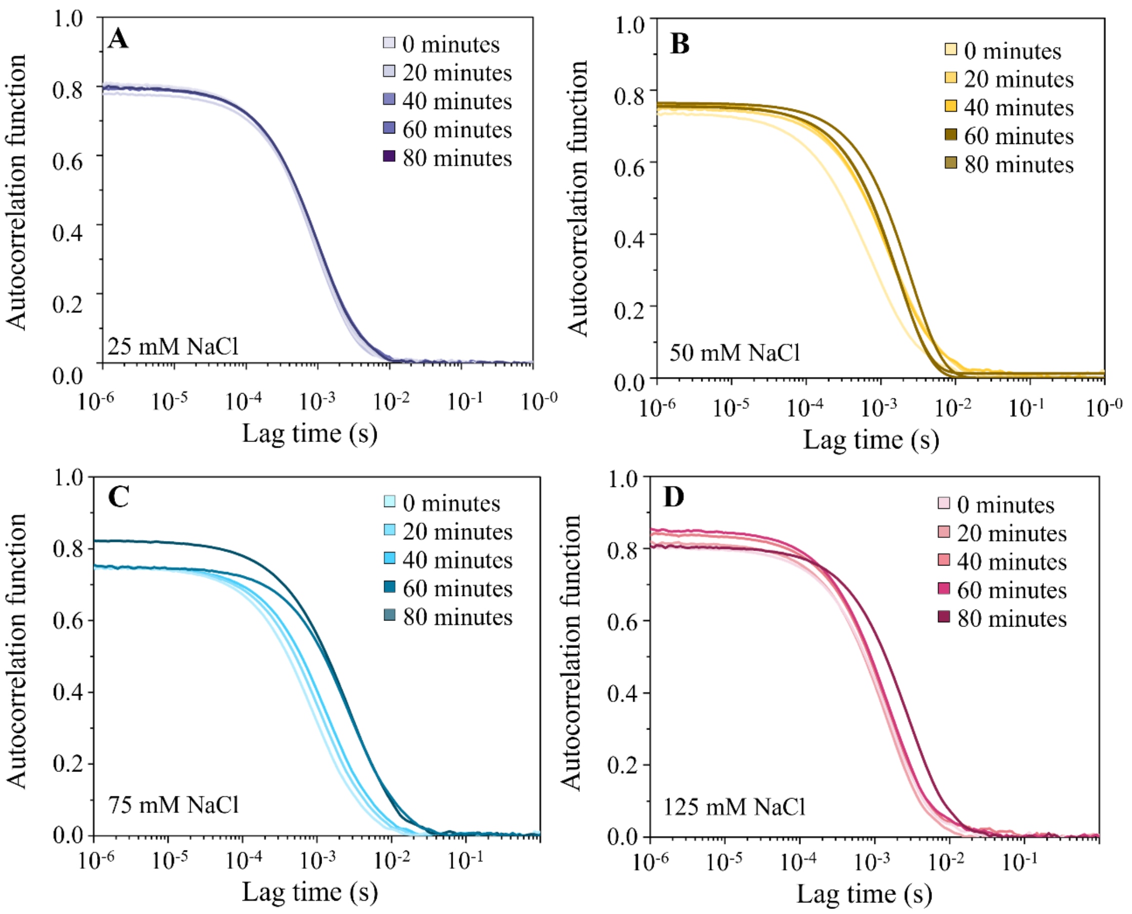

Figure S4: Autocorrelation functions (from DLS) of the fluvial sediment particles and aggregates at NaCl concentrations of (a) 25 mM, (b) 50 mM, (c) 75 mM, and (d) 125 mM over 80-minute time period. The measurements were fitted to a single exponential decay model, from which the size (effective diameter) of the fluvial sediment and aggregates were extracted.

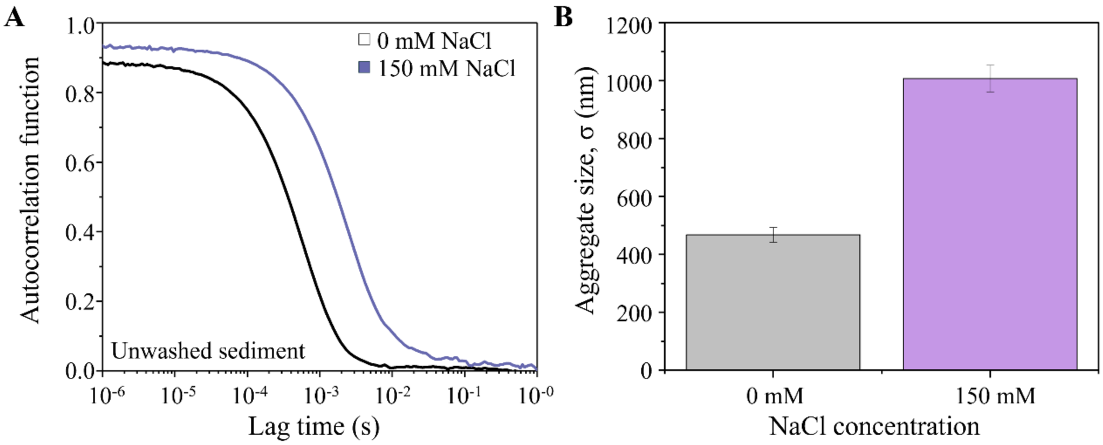

Figure S5: The size of the fluvial sediment and aggregates before washing. (a) Autocorrelation function of the unwashed sediment samples at 0 mM NaCl (black) and 150 mM NaCl (purple) obtained from dynamic light scattering (DLS). The measurements were performed after filtration though 0.45 µm syringe filter and 80 minutes of equilibration of sediments in water containing the respective NaCl concentrations. The autocorrelation functions were fit to a single exponential decay, from which the aggregate size was extracted. (b) The size of the sediment and sediment aggregates at 0 mM (grey) and 150 mM (purple) NaCl obtained from the fits of the autocorrelation function in (a). Here, the size increase by a factor of four, indicating that the mechanism presented in the manuscript holds true in the presence of organic matter.

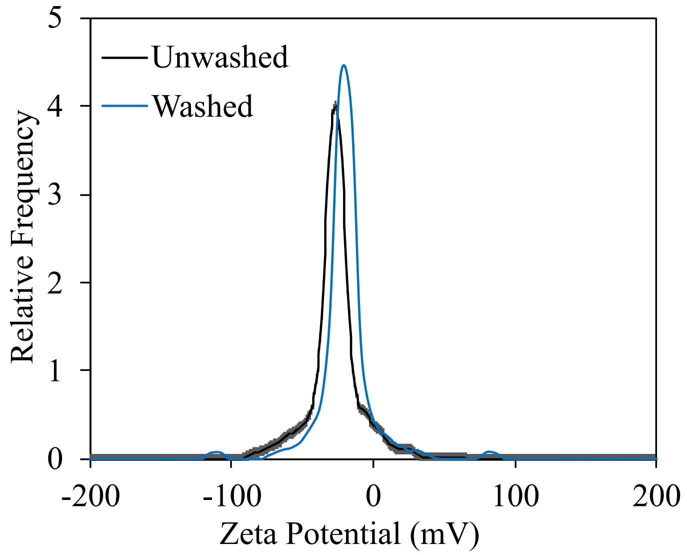

Figure S6: Relative charge distribution of the unwashed (black) and washed (blue) fluvial sediment. After treatment, the mean zeta-potential shifts from ~-25 mV to ~-20 mV.

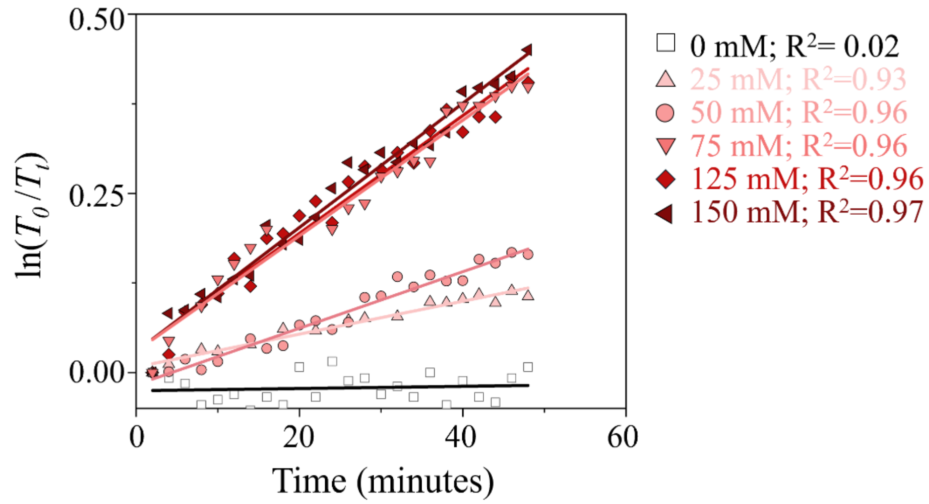

Figure S7: Variation of ln(T_0/T_t) as a function of time with respect to salinity. Symbols are experimental data, and lines are linear fits to the data. The R^2 values are presented in the legend as they were used as an indicator of pseudo-first order kinetics.

SALINITY	MODEL TYPE	KINETIC EQUATION	PARAMETERS	R^2	RATE CONSTANT, *k* (unit)
150 mM NaCl	Zero-order	$T_t = T_o - kt$	k, T_o	0.96	2.8×10^{-4} ($cm^2\,g^{-1}\,s^{-1}$)
	Pseudo-first-order	$\ln(T_t) = \ln(T_o) - kt$		0.97	1.7×10^{-4} (s^{-1})
	Second-order	$\frac{1}{T_t} = \frac{1}{T_o} + kt$		0.97	8.0×10^{-5} ($g\,cm^{-2}\,s^{-1}$)
75 mM NaCl	Zero-order	$T_t = T_o - kt$		0.88	2.0×10^{-4} ($cm^2\,g^{-1}\,s^{-1}$)
	Pseudo-first-order	$\ln(T_t) = \ln(T_o) - kt$		0.96	1.7×10^{-4} (s^{-1})
	Second-order	$\frac{1}{T_t} = \frac{1}{T_o} + kt$		0.96	4.5×10^{-5} ($g\,cm^{-2}\,s^{-1}$)
25 mM NaCl	Zero-order	$T_t = T_o - kt$		0.95	8.8×10^{-5} ($cm^2\,g^{-1}\,s^{-1}$)
	Pseudo-first-order	$\ln(T_t) = \ln(T_o) - kt$		0.93	4.7×10^{-5} (s^{-1})
	Second-order	$\frac{1}{T_t} = \frac{1}{T_o} + kt$		0.94	1.6×10^{-5} ($g\,cm^{-2}\,s^{-1}$)
0 mM NaCl	Zero-order	$T_t = T_o - kt$		0.01	1.0×10^{-6} ($cm^2\,g^{-1}\,s^{-1}$)
	Pseudo-first-order	$\ln(T_t) = \ln(T_o) - kt$		0.02	3.0×10^{-6} (s^{-1})
	Second-order	$\frac{1}{T_t} = \frac{1}{T_o} + kt$		0.02	1.0×10^{-6} ($g\,cm^{-2}\,s^{-1}$)

Table S1: Comparison of zero-order, pseudo-first-order, and second-order kinetic fits for turbidity decay at different NaCl concentrations. Rate constants (k) and coefficients of determination (R^2) were obtained by fitting turbidity (T) vs. time data to each kinetic model. Units of k reflect the form of each rate equation. While all three models yield similar R^2 values (>0.88), the pseudo-first-order model provides a satisfactory empirical description of the data. This comparison demonstrates that the observed trends in settling rate with salinity are robust to the choice of kinetic model.

Reference

1. Duduković, N.; Slijepčević, N.; Tomašević Pilipović, D.; Kerkez, Đ.; Krčmar, D., Synergistic Approaches for Enhanced Remediation of Polluted River Sediment. *Water, Air, & Soil Pollution* **2024,** *235* (6), 385.
2. Mun, Y.; Strmić Palinkaš, S.; Forwick, M.; Junttila, J.; Pedersen, K. B.; Sternal, B.; Neufeld, K.; Tibljaš, D.; Kullerud, K., Stability of Cu-Sulfides in Submarine Tailing Disposals: A Case Study from Repparfjorden, Northern Norway. *Minerals* **2020,** *10* (2), 169.
3. Draganits, E.; Gier, S.; Doneus, N.; Doneus, M., Geoarchaeological evaluation of the Roman topography and accessibility by sea of ancient Osor (Cres Island, Croatia). *Austrian Journal of Earth Sciences* **2019,** *112* (1), 1-19.
4. Twidwell, L. G.; Gammons, C. H.; Young, C. A.; Berg, R. B., Summary of Deepwater Sediment/Pore Water Characterization for the Metal-laden Berkeley Pit Lake in Butte, Montana. *Mine Water and the Environment* **2006,** *25* (2), 86-92.
5. Abdallah, A.; Kada, H.; Amrouche, A., Mechanical and thermo-physical characterization of mortars made with uncontaminated marine sediments. *Journal of Material Cycles and Waste Management* **2022,** *24* (6), 2483-2498.
